# Patient blood management in pregnancy, the peripartum, and the postpartum period

**DOI:** 10.61622/rbgo/2026FPS7

**Published:** 2026-06-03

**Authors:** Agnaldo Lopes da Silva, Álvaro Luiz Lage Alves, Gabriel Costa Osanan

**Affiliations:** Universidade Federal de Belo Horizonte Belo Horizonte MG Brazil Universidade Federal de Belo Horizonte, Belo Horizonte, MG, Brazil.; Universidade Federal de Belo Horizonte Belo Horizonte MG Brazil Universidade Federal de Belo Horizonte, Belo Horizonte, MG, Brazil.; Universidade Federal de Belo Horizonte Belo Horizonte MG Brazil Universidade Federal de Belo Horizonte, Belo Horizonte, MG, Brazil.

## Key points

- Obstetric patient blood management (PBM) marks a paradigm shift from reactive transfusion to proactive blood health management.
- Iron deficiency is the leading preventable cause of anemia in pregnancy, with significant maternal and neonatal consequences. Its detection requires measuring serum ferritin (below 30 µg/L) at the first prenatal visit.
- PBM is a multidisciplinary, evidence-based framework endorsed by the World Health Organization and organized around three pillars: optimization of erythropoiesis, minimization of blood loss, and optimization of tolerance to anemia.
- Intravenous iron is indicated when oral therapy fails or is not tolerated, or when rapid correction is required. Modern high-dose formulations allow complete repletion in one or two infusions.
- Tranexamic acid (1 g intravenously within 3 hours of postpartum hemorrhage onset) reduces bleeding-related death and should be integrated into all obstetric hemorrhage protocols.
- Fibrinogen levels below 2 g/L during active hemorrhage strongly predict progression to severe postpartum hemorrhage and necessitate immediate correction.
- PBM should be considered a core quality-of-care strategy in obstetric services, with the reduction of unnecessary transfusions serving as a key institutional performance indicator.

## Recommendations

- Screen all pregnant women for iron deficiency and anemia at the first prenatal visit using a complete blood count and serum ferritin; reassess at 24 to 28 weeks, after 32 weeks, and postpartum. *(Strong recommendation; moderate-quality evidence)*
- Initiate oral iron supplementation with 60 to 120 mg of elemental iron per day, or on alternate days, as first-line treatment for iron deficiency and mild iron-deficiency anemia *(strong recommendation, low-quality evidence*); switch to intravenous iron if hemoglobin increases by less than 1 g/dL after 2 to 4 weeks. *(Strong recommendation; high-quality evidence)*
- Offer intravenous iron to pregnant women after the first trimester of pregnancy and with intolerance or inadequate response to oral iron, hemoglobin below 9 g/dL, a diagnosis beyond 34 weeks or anemia requiring optimization before anticipated hemorrhage. Among available formulations, high-dose preparations allow complete repletion in fewer sessions. *(Strong recommendation; moderate-quality evidence)*. Intravenous iron should not be used in first trimester of pregnancy. (*Conditional recommendation; low-quality evidence)*
- Perform antenatal hemorrhage risk stratification and update it at admission for delivery, ensuring that all women identified as high risk deliver in facilities with immediate access to blood products, surgical expertise, and multidisciplinary teams. *(Strong recommendation; low-quality evidence)*
- Apply active management of the third stage of labor universally, using oxytocin (10 IU IM/IV) immediately after birth. *(Strong recommendation; high-quality evidence)*
- Administer tranexamic acid (1 g intravenously over 10 minutes) as early as possible in postpartum hemorrhage management and give a second dose after 30 minutes if bleeding persists or if it recurs within 24 hours of the initial administration. *(Strong recommendation; high-quality evidence)*
- Initiate hemostatic resuscitation when crystalloid infusion exceeds 1,500 to 2,000 mL without clinical response, targeting fibrinogen > 2 g/L, hemoglobin > 8 g/dL, and platelets > 50,000/mm^3^. *(Strong recommendation; moderate-quality evidence)*
- Apply restrictive transfusion thresholds (hemoglobin <7 g/dL) in hemodynamically stable postpartum women; consider higher thresholds (hemoglobin <8–9 g/dL) in the presence of ongoing bleeding, hemodynamic instability, or significant comorbidities. Transfusion decisions should be primarily guided by the overall clinical assessment rather than hemoglobin levels alone. Reassess after each unit transfused. *(Conditional recommendation; moderate-quality evidence)*
- In the setting of severe postpartum hemorrhage, early recognition of hypovolemic shock is critical. Physiological parameters such as the shock index can support timely decision-making, with values ≥1.0 associated with an increased likelihood of transfusion and clinical deterioration. *(Strong recommendation; low-quality evidence)*
- Implement institutional obstetric PBM protocols with multidisciplinary training, simulation-based hemorrhage drills, and regular audits of process and outcome indicators. *(Strong recommendation; low-quality evidence)*

## Background

Brazil remains among the countries with unacceptably high maternal mortality ratios, and obstetric hemorrhage is one of the leading direct causes of maternal death, with most fatalities considered preventable through timely recognition and systematic management.^(1,2)^ Iron deficiency anemia compounds the problem: approximately 20% to 40% of Brazilian pregnant women are anemic at some point during pregnancy, a prevalence that is higher in socioeconomically disadvantaged populations.^(3)^ Women who arrive at delivery with uncorrected anemia tolerate peripartum blood loss poorly, have higher transfusion rates, and experience worse postpartum recovery.^(1,2)^

Patient Blood Management (PBM), formally endorsed by the World Health Organization since 2010 (World Health Assembly resolution WHA63.12), offers a structured, proactive approach to these interconnected challenges.^(4,5)^ The Brazilian Federation of Gynecology and Obstetrics Associations (FEBRASGO) identifies the adoption of PBM principles across the continuum of pregnancy, delivery, and postpartum care as a strategic priority to improve maternal outcomes in Brazil. This Position Statement integrates and expands on the FEBRASGO 2024 Position Statement on the prevention and nonsurgical management of postpartum hemorrhage and the 2020 Position Statement on the surgical management of postpartum hemorrhage, providing comprehensive PBM guidance adapted to the Brazilian context.^(2,6)^

PBM is organized around three synergistic pillars, each corresponding to a specific phase of obstetric care (Chart 1).^(4,5,7)^ The first pillar, optimization of erythropoiesis, focuses on detecting and correcting iron deficiency and anemia before delivery, ensuring that every woman reaches the peripartum period with the best achievable hemoglobin concentration and iron stores. The second pillar, minimization of blood loss, encompasses all pharmacological, procedural, and surgical strategies that reduce hemorrhage during and after delivery, from active management of the third stage of labor to surgical hemostasis techniques. The third pillar, optimization of tolerance to anemia, addresses the rational use of blood products through restrictive transfusion thresholds, hemostatic resuscitation targets, and institutional protocols for decision-making, ensuring that transfusion is used when truly needed and avoided when the patient’s own compensatory mechanisms are sufficient.^(7,8)^

**Chart 1:** The three pillars of Patient Blood Management (PBM) applied to obstetric care

Pillar	Principle	Obstetric application	Timing
First	Optimization of erythropoiesis	Screening for iron deficiency and anemia; oral and intravenous iron therapy; folate supplementation	Prenatal and postpartum
Second	Minimization of blood loss	Active management of third stage; uterotonics; tranexamic acid; hemorrhage risk stratification; surgical hemostasis; cell salvage	Peripartum
Third	Optimization of tolerance to anemia	Restrictive transfusion thresholds for stable patients; hemostatic resuscitation targets; point of care testing; single unit policy with reassessment; Massive transfusion in the setting of severe hypovolemic shock.	Peripartum and postpartum

Sources: Shander et al. (2012),^(4)^ Shaylor et al. (2017)^(5)^ and Muñoz et al. (2018).^(7)^

## First pillar: optimization of erythropoiesis

### How should iron deficiency and anemia be screened and diagnosed during pregnancy?

Anemia in pregnancy is defined by the World Health Organization as hemoglobin below 11 g/dL in the first and third trimesters and below 10.5 g/dL in the second trimester.^(9,10)^ Iron deficiency is defined as serum ferritin below 30 µg/L, the most sensitive and specific single marker in pregnancy, even in the absence of anemia.^(11-13)^The physiological hemodilution that results from disproportionate plasma volume expansion relative to red cell mass can lower hemoglobin by 0.5 to 1.0 g/dL, but this should not be accepted as a reason to forgo investigation or treatment. Serum ferritin is an acute-phase reactant, and levels may be falsely normal or elevated in the presence of infection, inflammation, or preeclampsia. In such situations, transferrin saturation (below 20%) and C-reactive protein should be considered for interpretation.^(11,12)^ In routine practice, however, complete blood count and serum ferritin remain the most accessible and cost-effective screening tools. Chart 2 summarizes the recommended screening strategy for iron deficiency and anemia in pregnancy.

**Chart 2:** Recommended screening strategy for iron deficiency and anemia in pregnancy

Timing	Assessment
First prenatal visit (ideally first trimester)	Complete blood count and serum ferritin
24 to 28 weeks	Complete blood count; ferritin if risk factors present
> 32 weeks	Complete blood count and serum ferritin
Within 24 to 48 hours postpartum	Complete blood count; ferritin if antepartum anemia, peripartum hemorrhage, or symptoms
4 to 6 weeks postpartum	Complete blood count and serum ferritin if postpartum anemia or iron therapy ongoing

Risk factors include multiparity, short interpregnancy interval, adolescent pregnancy, vegetarian or vegan diet, multiple gestation, low socioeconomic status, history of menorrhagia, preeclampsia, and hematocrit below 30%. Iron deficiency: ferritin below 30 µg/L. Anemia: hemoglobin below 11 g/dL (first and third trimesters) or below 10.5 g/dL (second trimester)

Sources: OPAS (2018),^(1)^ Pavord et al. (2020)^(11)^ and Breymann et al. (2017).^(12)^

### What are the specific fetal and neonatal consequences of maternal iron deficiency and anemia?

The consequences of maternal iron deficiency and anemia extend significantly to the fetus and neonate. Iron is essential for fetal neurodevelopment, myelination, and hippocampal maturation; maternal iron deficiency, even without overt anemia, compromises transplacental iron transfer and depletes neonatal iron stores.^(14)^A systematic review and meta-analysis found that maternal anemia, particularly in the first and second trimesters, is independently associated with increased risk of preterm birth (OR 1.63; 95% CI 1.33–2.01), low birth weight (OR 1.31; 95% CI 1.13–1.51), and small-for-gestational-age neonates.^(15)^ Third-trimester anemia has been linked to increased risk of neonatal intensive care unit (NICU) admission and lower Apgar scores at 5 minutes.^(16)^ Neonatal iron deficiency, as a downstream consequence of inadequate maternal stores, is associated with impaired cognitive and psychomotor development, altered temperament, and deficits in attention and memory that may persist into school age even after iron repletion.^(14,17)^ These findings underscore the rationale for early, proactive screening and treatment of maternal iron deficiency, not solely for maternal benefit but also as a strategy to optimize neonatal outcomes and long-term child health.

### When and how should oral iron be prescribed?

Oral iron remains the recommended first-line treatment for iron deficiency and mild iron deficiency anemia in pregnancy (strong recommendation; high-quality evidence).^(9,11,12,18)^ Daily oral iron supplementation has consistently been associated with reduced maternal anemia at term, as demonstrated in a recent Cochrane systematic review, supporting current WHO recommendations for routine supplementation and treatment dosing.^(9,19)^The most commonly prescribed formulations are ferrous sulfate, ferrous fumarate, and ferrous gluconate. Recently, FIGO updated its recommendation for oral iron therapy, reducing the advised elemental iron dose for treating iron deficiency anemia. While earlier guidance recommended 100–200 mg/day, the current recommendation is 60–120 mg/day of elemental iron as first-line treatment, given as single daily doses on alternate days (as opposed to divided doses on consecutive days). This change is supported by evidence that oral iron rapidly increases hepcidin levels, which may remain elevated for up to 48 hours, thereby reducing the absorption of subsequent doses. In addition, lower-dose regimens have been shown to be as effective as higher doses for correcting iron deficiency anemia, while being better tolerated and associated with fewer gastrointestinal side effects.^(8)^Gastrointestinal side effects (nausea, constipation, abdominal pain) affect 30% to 50% of women and are the primary barrier to adherence.^(20)^Two randomized controlled trials demonstrated that alternate-day dosing improves fractional iron absorption via the hepcidin-mediated regulatory mechanism while reducing side effects, providing an evidence-based strategy to improve tolerability without compromising efficacy.^(21,22)^A recent systematic review by Kamath et al. found no significant difference in hemoglobin concentration between daily and alternate day oral iron administration, although alternate-day administration was associated with fewer gastrointestinal side effects.^(23)^ Treatment should be continued until the hemoglobin concentration reaches at least 11.0 g/dL. Thereafter, the dose should be reduced to a prophylactic regimen of 30–60 mg of elemental iron daily and maintained for 3 months or until 6 weeks postpartum, whichever is longer, in order to replenish iron stores.^(8)^ Sucrosomal iron is an innovative oral formulation in which ferric pyrophosphate is encapsulated within a phospholipid-sucrosome matrix, enabling absorption through M cells independently of the hepcidin-DMT-1 pathway and resulting in fewer gastrointestinal side effects than conventional ferrous salts.^(24,25)^Sucrosomial iron seems to be potentially effective and well tolerated at treating mild and moderate postpartum iron deficiency anemia.^(26)^Its use during pregnancy is currently being investigated. The expected response to adequate oral iron therapy is a hemoglobin increase of about 1 to 2 g/dL over 2 to 4 weeks. Failure to meet this target (a hemoglobin rise of less than 1 g/dL after 2 to 4 weeks) should prompt transition to intravenous iron.^(11,12)^

### What are the indications for intravenous iron in pregnancy and the postpartum period?

Intravenous iron is indicated in pregnant women beyond 14 weeks of gestation with(a) documented intolerance or nonadherence to oral iron; or (b) inadequate response to oral iron after 2 to 4 weeks of appropriate therapy; or (c) moderate to severe anemia (hemoglobin below 9 g/dL) at any gestational age; or (d) iron deficiency anemia diagnosed beyond 34 weeks, when time for oral correction is insufficient; or (e) anemia requiring optimization before anticipated peripartum hemorrhage, such as placenta previa, placenta accreta spectrum, or planned cesarean in women with anemia (strong recommendation; moderate quality evidence).^(8,11,12,18,27-29)^In the postpartum period, intravenous iron should be offered to women with hemoglobin below 9 g/dL, symptomatic anemia, or after significant peripartum hemorrhage, as it achieves faster repletion and is associated with more rapid improvement in hemoglobin, ferritin, and quality of life compared with oral iron.^(29-31)^Among available formulations, high-dose intravenous iron preparations allow complete iron repletion in one to two infusions, a decisive advantage in the obstetric setting, where time to correction before delivery is often limited.^(32,33)^Chart 3 compares the main intravenous iron formulations available for obstetric practice, including the maximum single dose, infusion time, and the number of sessions required for full repletion. All currently available formulations have a favorable safety profile, with rates of serious anaphylaxis well below 1 in 200,000 infusions.^(34)^ Transient hypophosphatemia has been reported more often with some formulations, though it is usually asymptomatic after a single infusion.^(35)^ A systematic review and meta-analysis confirmed the superiority of intravenous iron over oral iron for correcting anemia in pregnancy, with faster hemoglobin recovery and comparable safety.^(36)^

**Chart 3:** Intravenous iron formulations available for obstetric practice

Parameter	Iron sucrose	Ferric carboxymaltose	Ferric derisomaltose
Maximum single dose	200 mg	1,000 mg (up to 20 mg/kg depending on regional labeling)	Up to 20 mg/kg (typically up to 1500 mg)
Infusion time (single dose)	30 to 60 minutes	15 to 30 minutes	20 to 60 minutes
Number of infusions for full repletion (typical 1,000 to 1,500 mg deficit)	5 to 8 sessions	1 to 2 sessions	1 session
Test dose required	No	No	No
Gestational age for use	Preferably from 2nd trimester	Preferably from 2nd trimester	Preferably from 2nd trimester
Risk of hypophosphatemia	Low	Higher (transient; may be clinically relevant)	Low
Serious anaphylaxis risk	Very low (rare)	Very low (rare)	Very low (rare)

All formulations are generally avoided in the first trimester due to limited safety data. The iron deficit can be estimated by the Ganzoni formula or a simplified weight-based calculation. The choice among formulations should be guided by local availability, cost, and clinical judgment. Ganzoni formula for total iron deficit calculation:  Total iron dose (mg)= body weight (kg)×[ target Hb− actual Hb](g/dL]×2.4+ iron stores (usually 500−1000mg) . Target hemoglobin: 11 g/dL during pregnancy and 12 g/dL in the postpartum period. In women with obesity, consider using ideal body weight or pre-pregnancy weight for dose calculation

Sources: Muñoz et al. (2018),^(7)^ Auerbach and Macdougall (2017),^(32)^ Govindappagari and Burwick (2019)^(36)^ and ABHH (2023)^(37)^

**Figure 1:**
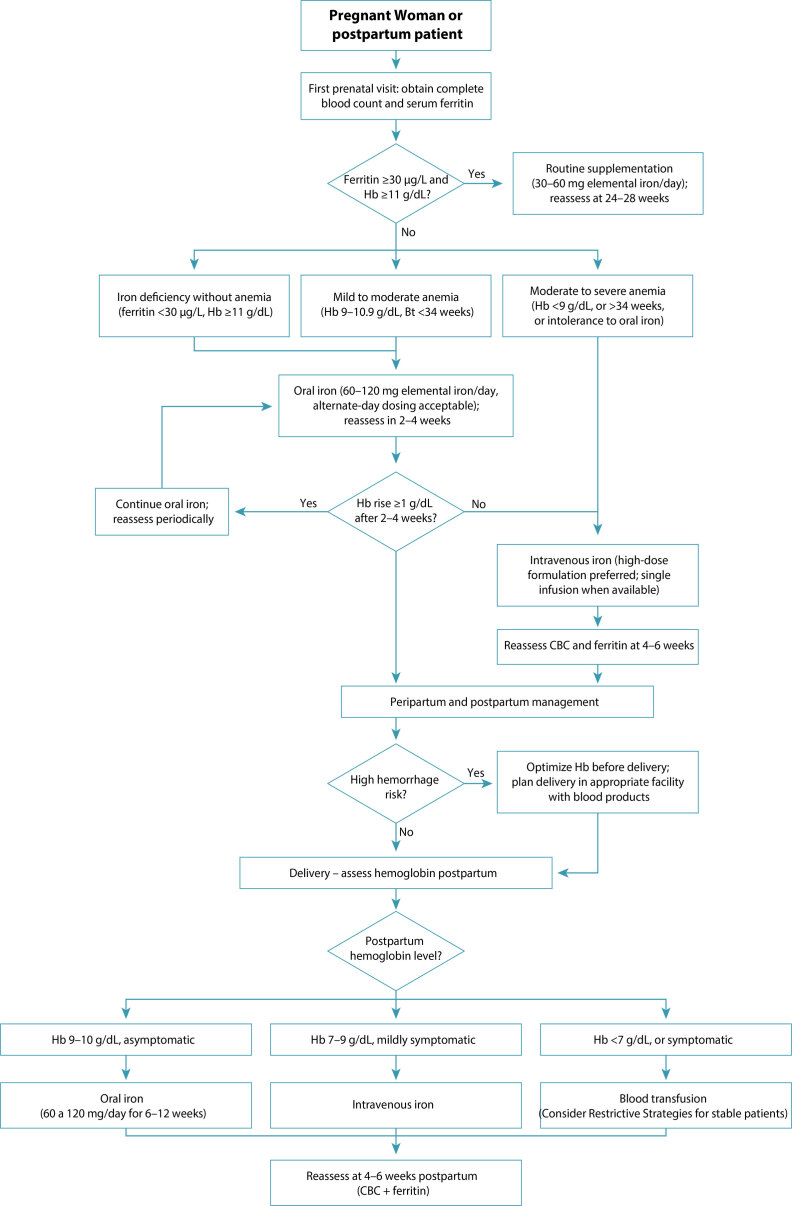
Algorithm for the diagnosis and treatment of iron deficiency and iron deficiency anemia in pregnancy and the postpartum period Clinical algorithm for the screening, diagnosis, and stepwise management of iron deficiency and iron deficiency anemia across pregnancy and the postpartum period. Screening with complete blood count and serum ferritin is recommended at the first prenatal visit, 24–28 weeks, > 32 weeks, and within 48 hours postpartum. Treatment decisions are guided by gestational age, hemoglobin and ferritin levels, symptom severity, and response to initial oral therapy. The threshold for transition to intravenous iron is a hemoglobin rise of less than 1 g/dL after 2 to 4 weeks of oral supplementation. CBC: complete blood count; Hb: hemoglobin; ID: iron deficiency; IDA: iron deficiency anemia; IV: intravenous.

Sources: Muñoz et al. (2018),^(7)^ Pavord et al. (2020)^(11)^ and Breymann et al. (2017).^(12)^

All women with postpartum anemia or recent postpartum hemorrhage should undergo a complete blood count and have ferritin reassessed at 4 to 6 weeks postpartum to confirm adequate recovery.^(7,30)^Figure 1 integrates the screening, diagnosis, and treatment steps described above into a single clinical algorithm for managing iron deficiency and iron deficiency anemia throughout pregnancy and the postpartum period. This algorithm is intended as a practical decision-support tool for prenatal, labor and delivery, and postpartum care teams. Women need to be monitored during intravenous iron infusion and for at least 30 minutes after infusion for anaphylactic reactions.^(10)^

## Second pillar: minimization of blood loss

### How should hemorrhage risk be stratified during pregnancy and at admission?

Antenatal risk stratification is a cornerstone of obstetric PBM and should be performed throughout prenatal visits and updated at delivery admission (strong recommendation; low-quality evidence).^(1,2,7)^ The risk factors for severe PPH include previous PPH, placenta previa or accreta spectrum, preeclampsia with signs of severity, hematocrit below 30%, platelet count below 100,000/mm^3^, active bleeding on admission, coagulopathies, and anticoagulant use.^(1,2)^ Every pregnant woman with a prior cesarean section should undergo ultrasonography to determine placental location; in cases of placenta previa or implantation in the uterine segment, investigation for signs of placenta accreta and referral to a tertiary center are indicated.^(1,6)^Chart 4 presents a hemorrhage risk stratification framework adapted to the obstetric setting.

**Chart 4:** Risk stratification for postpartum hemorrhage

Risk category	Criteria	Recommended actions
Low	No prior uterine surgery, singleton, four or fewer previous vaginal deliveries, no history of PPH, no bleeding disorder, hemoglobin 11 g/dL or above	Blood type and screen; active management of third stage; oxytocin prophylaxis
Medium	Prior cesarean or uterine surgery, more than four previous deliveries, multiple gestation, large fibroids, history of PPH, chorioamnionitis, hemoglobin 8 to 10.9 g/dL	Cross match; ensure large bore intravenous access; uterotonics available; notify anesthesia and blood bank
High	Placenta previa or accreta spectrum, hemoglobin below 8 g/dL, platelets below 100,000/mm^3^, active bleeding on admission, known coagulopathy, anticoagulant use, placental abruption, preeclampsia with severity features	Cross matched blood available; multidisciplinary team alert (obstetricians, anesthesiologists, blood bank, interventional radiology); tertiary level care; cell salvage if available

PPH, postpartum hemorrhage

Sources: OPAS (2018),^(1)^ Alves et al. (2024),^(2)^ Muñoz et al. (2018)^(7)^ and Main et al. (2015)^(38)^

### What pharmacological strategies minimize peripartum blood loss?

#### Active management of the third stage and uterotonics

Active management of the third stage of labor, comprising prophylactic uterotonics, timely cord clamping (between one and three minutes), and controlled cord traction, is the most effective single intervention for PPH prevention and should be applied universally (strong recommendation; high-quality evidence).^(1,2,39)^ The use of a quality-assured uterotonic is recommended for the prevention of postpartum haemorrhage during the third stage of labour for all births. To effectively prevent postpartum haemorrhage, one of the following uterotonics should be used: oxytocin, carbetocin and misoprostol. Ergometrine/methylergometrine is no longer recommended for the prevention of postpartum haemorrhage, but it remains an important therapeutic option for treatment of uterine atony. In settings where multiple uterotonic options are available, oxytocin (intramuscularly/intravenously) is the recommended uterotonic agent of choice. If oxytocin is unavailable or its quality cannot be guaranteed, heat-stable carbetocin or misoprostol is recommended.^(1,2,40)^ The CHAMPION trial showed that heat-stable carbetocin (100 µg intramuscularly) is noninferior to oxytocin and offers the advantages of thermostability and a longer-lasting uterotonic effect.^(41)^

#### Tranexamic acid

The WOMAN trial (n = 20,060) demonstrated that tranexamic acid (1 g intravenously within 3 hours of PPH onset) significantly reduced death due to bleeding (relative risk 0.81; 95% confidence interval 0.65 to 1.00), with no increase in thromboembolic events (strong recommendation; high quality evidence).^(42)^ Tranexamic acid should be administered intravenously as early as possible at a dose of 1 gram in 100 mL of 0.9% saline over 10 minutes. A second dose may be given after 30 minutes if bleeding persists or if it recurs within 24 hours. Administration beyond 3 hours from PPH onset appears to offer no benefit.^(1,2)^

#### Intraoperative cell salvage

Intraoperative cell salvage with leukocyte-depletion filters is safe and effective in obstetric surgery. It is recommended for anticipated major hemorrhage, such as placenta accreta spectrum, which requires a multidisciplinary team and tertiary-level care (conditional recommendation; moderate-quality evidence).^(6,43)^ Cell salvage reduces the need for allogeneic transfusions while avoiding the immunological and infectious risks associated with donor blood. The SALVO trial confirmed the safety and feasibility of cell salvage during cesarean delivery, although routine use in all cesareans was not supported by cost-effectiveness analysis.^(43)^ More recent guidance from international organizations, including WHO and FIGO (2025), does not support the routine use of cell salvage in obstetrics outside of well-designed research settings, highlighting the need for further studies, particularly in populations at high risk of hemorrhage, such as placenta accreta spectrum. In this context, its use should be considered investigational, ideally within structured protocols and multidisciplinary settings.^(8,40)^Chart 5 summarizes the pharmacological strategies for minimizing peripartum blood loss.

**Chart 5:** Pharmacological and procedural strategies for peripartum blood loss minimization

Strategy	Indication	Dose and route	Strength of recommendation; quality of evidence	Key evidence
Oxytocin	Prevention (universal) and treatment of atony	10 IU IM/IV for vaginal delivery (prevention); 10 or 5 IU slow IV bolus followed by infusion (treatment)	Strong; high	OPAS (2018),^(1)^ WHO (2025)^(40)^
Heat stable carbetocin	Only for Prevention (alternative to oxytocin)	100 µg IM, single dose	Strong; high	Widmer et al. (2018)^(41)^
Tranexamic acid	Treatment of PPH;	1 g IV over 10 minutes; repeat after 30 minutes if needed	Strong; high	WHO (2025),^(40)^ WOMAN (2017)^(42)^
Methylergometrine	Second line uterotonic for treatment. No longer used for prevention (contraindicated in hypertension)	0.2 mg IM; may repeat after 20 minutes	Strong; moderate	OPAS (2018)^(1)^ WHO (2024)^(10)^
Misoprostol	Second or third line uterotonic for prevention and treatment	800 to 1,000 µg rectally *	Strong; moderate	OPAS (2018),^(1)^
Intraoperative cell salvage	Anticipated major hemorrhage (placenta accreta spectrum)	Per institutional protocol	Conditional; moderate	Khan et al. (2017)^(43)^

*Oral formulation of misoprostol is not available in Brazil. IU, international units; PPH, postpartum hemorrhage; IM, intramuscular; IV, intravenous

## What is the role of the shock index and the golden hour concept in postpartum hemorrhage management?

**Figure 2:**
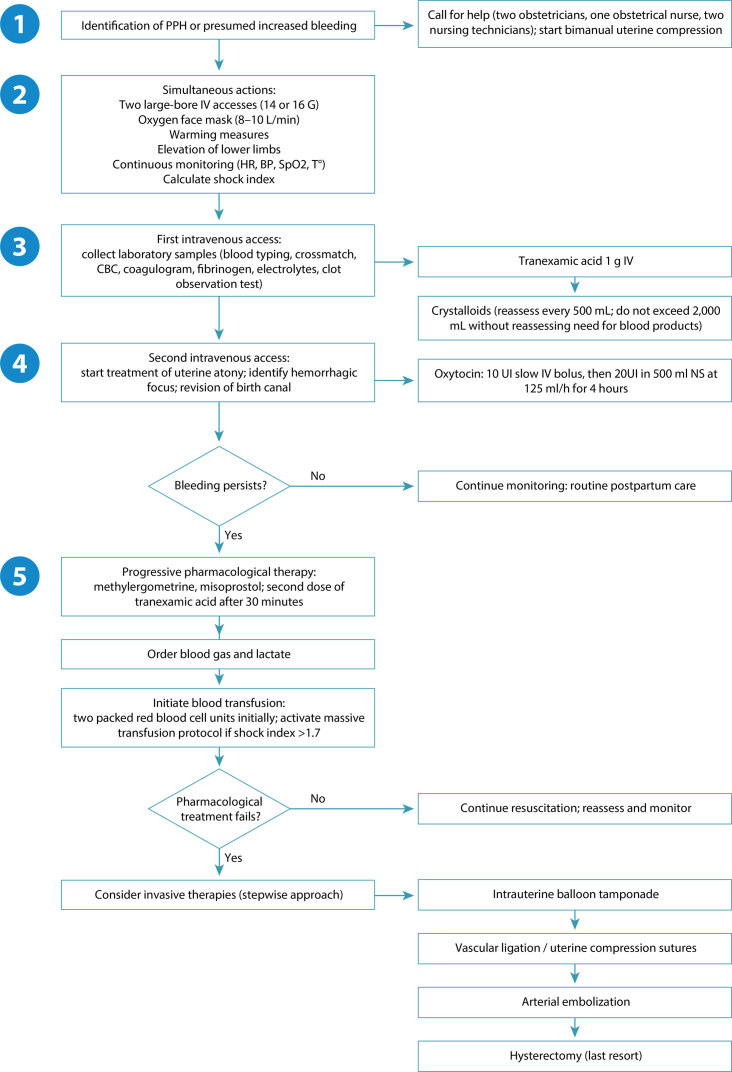
Flowchart for initial management of postpartum hemorrhage Stepwise protocol for the early recognition and integrated management of postpartum hemorrhage based on Patient Blood Management principles. The algorithm outlines five sequential phases: identification and call for help (Step 1), simultaneous resuscitation measures and Shock Index calculation (Step 2), laboratory sampling with early tranexamic acid and crystalloid infusion (Step 3), source-directed treatment with uterotonics (Step 4), and escalation to hemostatic resuscitation, blood transfusion, massive transfusion protocol activation, and invasive surgical therapies when pharmacological treatment fails (Step 5). SI: Shock Index; TXA: tranexamic acid; PPH: postpartum hemorrhage; MTP: massive transfusion protocol; IV: intravenous; Hb: hemoglobin. Note: PPH, postpartum hemorrhage

Sources: OPAS (2018),^(1)^ Alves et al. (2024)^(2)^ and Alves et al. (2020)^(6)^

The concept of the “golden hour in obstetrics” refers to controlling the hemorrhagic source within the first hour after diagnosis, which is the most effective strategy to prevent progression to hypovolemic shock^(1,2)^ Through early, aggressive, efficient, and organized management, the lethal triad of hemorrhagic shock (hypothermia, acidosis, and coagulopathy) can be avoided.^(1,6)^The shock index (heart rate divided by systolic blood pressure) is the recommended clinical method for estimating blood loss and predicting the need for transfusion. Values of 0.9 or above indicate significant blood loss. Values of 1.0 or above signal the need for rapid intervention and possible transfusion. Values between 1.3 and 1.7 suggest moderate shock, and values above 1.7 indicate severe shock with potential need for massive transfusion.^(1,2)^Figure 2 presents the initial clinical management algorithm for postpartum hemorrhage.

## When is urgent anticoagulation reversal required, and what is the role of prothrombin complex concentrate (PCC) in obstetric hemorrhage?

The increasing use of prophylactic and therapeutic anticoagulation during pregnancy, particularly low-molecular-weight heparins (LMWH) and, less commonly, vitamin K antagonists (VKA), creates clinical scenarios in which emergent reversal is required to achieve surgical hemostasis or control PPH.^(44)^ PBM frameworks mandate that reversal strategies be predefined in institutional massive transfusion protocols (MTP) to avoid delays in critical bleeding.^(7)^For LMWH, protamine sulfate achieves only partial neutralization (approximately 60–80% of anti-Xa activity), and its role remains limited. For VKA-associated coagulopathy, intravenous phytonadione (vitamin K₁, 10 mg) provides definitive but delayed reversal (12–24 hours), making it insufficient as a sole strategy during active hemorrhage.^(45)^Four-factor prothrombin complex concentrate (4F-PCC) contains factors II, VII, IX, and X, along with proteins C and S, and enables rapid, volume-efficient correction of VKA-induced coagulopathy. Compared with fresh frozen plasma (FFP), 4F-PCC provides faster INR normalization (within 15–30 minutes vs. hours), a substantially smaller infusion volume (typically 20–40 mL vs. 600–900 mL for equivalent correction), a lower risk of transfusion-associated circulatory overload (TACO), and no need for ABO compatibility or thawing time.^(46,47)^ These advantages are particularly relevant in the obstetric setting, where rapid hemostatic correction and avoidance of volume overload are paramount.

Although direct evidence in obstetric populations is limited, data extrapolated from trauma, cardiac surgery, and perioperative medicine consistently demonstrate the efficacy and safety of 4F-PCC in reversing acute coagulopathy.^(45,48)^ The European Society of Anaesthesiology (ESA) guidelines recommend PCC over FFP for rapid VKA reversal in the context of life-threatening hemorrhage (Grade 1B).^(45)^ Its use may also be considered in acquired coagulopathy with a documented factor deficiency beyond VKA exposure, although this remains an off-label application requiring individualized assessment and guidance based on viscoelastic testing (TEG/ROTEM).^(49)^Institutions implementing PBM programs should ensure 4F-PCC availability in labor and delivery units, establish weight-based dosing protocols stratified by baseline INR, and include PCC in simulation-based MTP drills (Strong recommendation; low certainty of evidence - extrapolated).

## Should cryoprecipitate or fibrinogen concentrate be used for fibrinogen replacement in obstetric hemorrhage?

Hypofibrinogenemia is an early and rapidly evolving feature of severe postpartum hemorrhage. Two sources of exogenous fibrinogen are available for replacement therapy in obstetric hemorrhage: cryoprecipitate and fibrinogen concentrate.^(50)^ Understanding their comparative profiles is essential for designing institutional protocols. Cryoprecipitate is derived from thawed FFP and contains fibrinogen (approximately 250 mg per unit, variable), factor VIII, factor XIII, von Willebrand factor, and fibronectin. Standard adult dosing requires pooling 8–10 units to achieve a meaningful increment (approximately 0.5–1.0 g/L rise per 10 units). Key limitations include the need for ABO-compatible selection, thawing time (20–30 minutes), batch-to-batch variability in fibrinogen content, and a residual, albeit low, risk of transfusion-transmitted infection, TACO, and transfusion-related acute lung injury (TRALI).^(49,51)^Fibrinogen concentrate is a purified, lyophilized, pathogen-inactivated product with standardized fibrinogen content (typically 1 g per vial), reconstituted in a small volume (50 mL), and administered without thawing, cross-matching, or ABO compatibility testing. Time from decision to infusion is substantially shorter (5–10 minutes vs. 30–60 minutes for cryoprecipitate), and dosing precision is superior.^(47,50)^ The OBS2 trial demonstrated that empirical early fibrinogen concentrate (2 g) in PPH was feasible and well-tolerated, though it did not reduce allogeneic transfusion in the overall cohort when administered without fibrinogen-level guidance.^(49)^ Current ESA guidelines and the ISTH consensus recommend fibrinogen replacement when plasma fibrinogen falls below 2 g/L (or FIBTEM A5 < 12 mm on ROTEM), with an initial dose of 25–50 mg/kg (approximately 2-4 g).^(45,49)^ The choice between cryoprecipitate and fibrinogen concentrate should be guided by local availability, cost, logistics (including blood bank proximity and thawing infrastructure), and patient-specific factors. In settings where rapid administration is critical and blood bank logistics are challenging, fibrinogen concentrate provides logistical advantages.

## Third pillar: optimization of tolerance to anemia

### How should hemostatic resuscitation and transfusion be managed during obstetric hemorrhage?

#### Goal directed hemostatic therapy

Obstetric coagulopathy develops early in PPH and is characterized by rapid fibrinogen consumption. Fibrinogen normally is elevated in the third trimester (4 to 6 g/L).^(53)^ Fibrinogen levels below 2 g/L have a 100% positive predictive value for progression to severe PPH, making early measurement and aggressive correction of hypofibrinogenemia essential (strong recommendation; moderate-quality evidence).^(1,45,53)^Point of care viscoelastic hemostatic assays (thromboelastography and rotational thromboelastometry) provide rapid assessment of clot formation and guide targeted correction of specific hemostatic deficits when available (conditional recommendation; moderate quality evidence).^(54)^ When unavailable, clinical assessment and standard laboratory tests, especially fibrinogen, coagulogram, platelet count, and the clot observation test, should guide replacement therapy.^(1)^Fibrinogen concentrate offers a more targeted, rapid, and volume-efficient approach than fresh frozen plasma for correcting hypofibrinogenemia during PPH.^(45,55)^ Platelet levels should be maintained above 100,000/mm^3^ during active bleeding and above 50,000/mm^3^ after bleeding has been controlled.^(1)^

#### Transfusion decisions

Volume resuscitation with crystalloids should be assessed every 500 mL infused and generally should not, exceed a total of 2,000 mL. Rapid and excessive crystalloid infusion can paradoxically increase bleeding, favor hypothermia, and dilute coagulation factors, advancing the lethal triad.^(1,2)^ After infusion of 1,500 mL of crystalloids in the presence of active bleeding, hemodynamically unstable patients (or those with partial hemodynamic response) should be evaluated for immediate blood transfusion. In acute haemorrhage, initial transfusion should be guided primarily by clinical signs of maternal instability. Hemoglobin and hematocrit have limited value in acute bleeding, as they may take 2 to 4 hours to reflect the magnitude of blood loss.^(1,2)^ In this context, the shock index, a marker of hemodynamic instability, is a valuable tool for predicting the need for blood transfusion. Obstetrics transfusion protocol should be available in all birth care units. Hemodynamically unstable patients with significant losses should receive emergency transfusion of two packed red blood cell units. If cross-matching is unavailable, O-negative blood should be transfused.^(1,2)^ In the face of severe shock (shock index>1.7), massive transfusion should be initiated, immediately, preferably with balanced proportions of packed red blood cells, fresh-frozen plasma, cryoprecipitate, and platelets and based in a massive transfusion protocol.^(1)^Restrictive transfusion strategies (a hemoglobin threshold below 7 g/dL in hemodynamically stable patients) are safe and noninferior to liberal strategies (conditional recommendation; moderate-quality evidence).^(56)^ In the obstetric setting, transfusion should be considered at hemoglobin levels below 7 g/dL in hemodynamically stable postpartum women, or at higher thresholds (hemoglobin levels below 8 to 9 g/dL) in the presence of ongoing bleeding, hemodynamic instability, or significant comorbidities.^(5,7)^ A single unit policy, with clinical reassessment after each unit, avoids unnecessary exposure. Chart 6 summarizes the hemostatic resuscitation targets during major obstetric hemorrhage.

**Chart 6:** Targets for hemostatic resuscitation in major obstetric hemorrhage

Parameter	Target
Hemoglobin	Above 8 g/dL
Fibrinogen	Above 2 g/L (above 200 mg/dL)
Platelet count	Above 50,000/mm^3^ (or above 100,000/mm^3^ if active bleeding)
International normalized ratio	1.5 or below
Temperature	Above 35 °C (avoid hypothermia)
Ph	>7,2
Base excess	<-6 mmol/L
Lactato	<2 mmol/L
Ionized calcium	Above 1.0 mmol/L

These targets should be interpreted in conjunction with the clinical status, the rate and pattern of bleeding, and point of care test results when available

Sources: OPAS (2018),^(1)^ Alves et al. (2024)^(2)^ and Kozek-Langenecker et al. (2017)^(45)^

## How can oxygen delivery be maximized and oxygen consumption reduced in the anemic obstetric patient?

The third pillar of PBM extends beyond transfusion thresholds to include the physiological optimization of the oxygen supply-demand balance. This approach is particularly relevant when allogeneic transfusion is unavailable, delayed, or declined by the patient.^(57)^

### Maximizing oxygen delivery

Supplemental oxygen therapy should be titrated to maintain arterial oxygen saturation (SpO₂) ≥ 95%In mechanically ventilated patients, optimizing FiO₂ and positive end-expiratory pressure (PEEP) improves arterial oxygen content independently of hemoglobin concentration. Maintaining adequate intravascular volume, avoiding both hypovolemia and hypervolemia, ensures optimal cardiac output and tissue perfusion.^(57,58)^ Vasopressors (norepinephrine) should be considered early in distributive shock to maintain mean arterial pressure (MAP) ≥ 65 mmHg and preserve coronary and uterine perfusion.^(7)^

### Reducing oxygen consumption

Pain, anxiety, shivering, fever, hypothermia and infection can significantly raise metabolic demand, especially during the peripartum period. Implementing effective multimodal analgesia, using neuraxial techniques, when possible, intravenous paracetamol, and careful opioid use, helps lessen sympathetic nervous system activation and oxygen use. Active temperature regulation including warmed IV fluids, forced-air warming blankets, and maintaining ambient temperature, helps prevent hypothermia-related coagulopathy and reduces shivering-induced increases in VO₂, which can reach 200–300%.^(45,59)^ Additionally, treating infections and sepsis promptly with suitable antibiotics decreases the hypermetabolic response, thereby improving tolerance to anemia. These measures, although individually supported by moderate-to-low certainty evidence in obstetric populations, are physiologically sound, low cost, and universally implementable. They should be incorporated into every institutional PBM protocol as standard adjunctive care for the anemic or hemorrhaging obstetric patient.

## Which clinical factors impair physiological tolerance to anemia and should prompt a higher transfusion threshold?

Not all patients tolerate the same degree of anemia equally. Several clinical conditions impair the compensatory mechanisms (increased cardiac output, enhanced oxygen extraction, leftward shift avoidance) that maintain tissue oxygenation during normovolemic anemia. Recognizing these factors is critical for individualizing transfusion decisions beyond a single hemoglobin threshold.^(58,60)^

Factors that reduce anemia tolerance in the obstetric patient include:

- Pre-existing cardiovascular disease: including valvular heart disease, cardiomyopathy, and pulmonary hypertension, that limits the compensatory increase in cardiac output;^(60)^
- Acute hemodynamic instability: tachycardia (HR > 100 bpm), hypotension (MAP < 65 mmHg), or elevated Shock Index (≥ 0.9), indicating depleted cardiovascular reserve;^(61)^
- Ongoing or uncontrolled hemorrhage, where the trajectory of hemoglobin decline matters more than a single static value;^(7)^
- Sepsis and systemic infection, which increase metabolic demand and impair microcirculatory oxygen extraction;^(62)^
- Fever and hyperthermia, raising oxygen consumption by approximately 10% per degree Celsius above 37°C;^(45)^
- Hypoxemia from any cause (respiratory disease, airway compromise, high altitude), which compounds the reduction in arterial oxygen content caused by anemia;^(58)^
- Pre-eclampsia and eclampsia, where endothelial dysfunction and microvascular disease impair tissue oxygen delivery independently of hemoglobin;
- Maternal obesity and obstructive sleep apnea, which predispose to both hypoxemia and reduced functional residual capacity.

In the presence of one or more of these factors, a higher transfusion threshold(Hb < 8–9 g/dL rather than < 7 g/dL) may be appropriate, guided by clinical assessment and, when available, markers of tissue perfusion (lactate, ScvO₂, urine output).

## How should postpartum anemia be managed?

Postpartum anemia (hemoglobin below 10 g/dL within 48 hours of delivery) affects up to 50% of women in some populations and is associated with fatigue, impaired lactation, postpartum depression, and delayed functional recovery.^(14,30)^ Women with mild postpartum anemia (hemoglobin 9 to 10 g/dL) who are asymptomatic may be treated with oral iron (60 to 120 mg of elemental iron daily for 6 to 12 weeks) (strong recommendation; moderate-quality evidence).^(30)^ Women with moderate-to-severe anemia (hemoglobin below 9 g/dL), particularly if symptomatic or following significant hemorrhage, should be offered intravenous iron, as randomized controlled trials have consistently shown faster recovery of hemoglobin and ferritin and better quality-of-life outcomes than oral iron (strong recommendation; moderate-quality evidence).^(31)^ All women with postpartum anemia should have a complete blood count and ferritin reassessed at 4 to 6 weeks postpartum to confirm adequate recovery.^(30)^

## How should transfusion governance be structured within institutional PBM programs?

The sustainability of PBM initiatives requires formal integration into the institution’s transfusion governance framework. A multidisciplinary transfusion committee, comprising obstetricians, anesthesiologists, hematologists, transfusion medicine specialists, nursing leadership, and hospital pharmacy, should be responsible for:^(7)^

- Developing and periodically updating institutional obstetrics transfusion guidelines aligned with PBM principles;
- Reviewing transfusion appropriateness through prospective audit and feedback cycles, including analysis of single-unit compliance, pre-transfusion hemoglobin documentation, and clinical indication recording;
- Conducting hemovigilance: systematic monitoring of adverse transfusion reactions (acute and delayed), near-miss events, and tracking transfusion-related outcomes in obstetric patients;
- Ensuring blood inventory optimization for labor and delivery units, including type-and-screen policies for patients at risk, pre-positioned emergency O-negative units, and massive transfusion pack logistics;
- Reporting institutional transfusion metrics to hospital leadership and, where applicable, to national hemovigilance systems.

Regular simulation-based massive transfusion protocol (MTP) drills, conducted at least biannually and involving the full multidisciplinary team, have been shown to reduce time-to-blood-delivery, improve team communication, and decrease protocol deviations during real events.^(63,64)^ Each drill should be followed by structured debriefing with documentation of performance gaps and targeted improvement actions. Institutions with established PBM governance have demonstrated 15–30% reductions in allogeneic red blood cell transfusion rates, decreased length of stay, and lower transfusion-associated complication rates across surgical and obstetric populations.^(65)^

## Implementation

### How should institutional PBM programs be implemented in maternity units?

The successful implementation of PBM in obstetric care requires an institutional, multidisciplinary approach (strong recommendation; low quality evidence).^(1,2,5-7,66)^ Key components include:

- Development of evidence-based PBM protocols and clinical pathways integrated into existing maternal safety bundles and obstetric early warning systems.
- Multidisciplinary engagement including obstetricians, anesthesiologists, hematologists, blood bank professionals, nursing, and midwifery.
- Simulation-based training for PPH management with defined roles, including skills for surgical hemorrhage control techniques such as vascular ligation and uterine compression sutures.^(6)^
- Hemorrhage risk stratification at every admission for delivery, as described in chart 6.
- Availability of PPH management kits accessible to the entire care team.
- Audit and feedback cycles to monitor key PBM indicators, as proposed in chart 7.
- Organization of the healthcare network to guarantee care flows and access to more complex treatments, including availability of blood products, intravenous iron, cell salvage, and referral pathways.

Chart 7 outlines quality indicators for obstetric PBM programs, and chart 8 provides a practical implementation checklist for maternity units.

Integrating PBM into existing PPH prevention and treatment protocols, as recommended by OPAS and FEBRASGO, represents a natural and efficient implementation strategy.^(1,2)^ The emphasis on reducing maternal mortality from hemorrhage in Brazil, including the Strategy for Zero Maternal Death by Hemorrhage initiative, aligns with the PBM framework.^(67)^Surgical conservative techniques for hemorrhage control should be part of the institutional skill set and applied before hysterectomy when pharmacological or other less invasive treatment fails or are not indicated.^(6)^

## Final considerations

In Brazil, implementing obstetric PBM faces specific challenges: heterogeneous availability of serum ferritin testing across the public healthcare system (SUS); limited availability of high-dose intravenous iron formulations in many public institutions; and variable access to cell salvage technology and point-of-care hemostatic testing. Nonetheless, the core principles of PBM are applicable at all levels of care and can be implemented incrementally. PBM marks a paradigm shift from reactive, transfusion-based approaches to a proactive, preventive, patient-centered strategy. In obstetrics, the three pillars of PBM provide a comprehensive framework for addressing the interconnected challenges of iron deficiency, anemia, and hemorrhage-related morbidity and mortality. Early identification and treatment of iron deficiency, with timely use of intravenous iron when indicated, is a fundamental, cost-effective intervention that optimizes maternal hemoglobin levels before delivery and accelerates postpartum recovery. Systematic hemorrhage risk stratification, evidence-based use of uterotonics and tranexamic acid, prompt bleeding control within the golden hour, goal-directed hemostatic management, and restrictive transfusion strategies complete the framework. FEBRASGO urges all maternity units in Brazil to adopt PBM principles as an integral component of comprehensive, safe obstetric care, supported by institutional protocols, multidisciplinary training, regular audits, and continuous quality improvement.

## References

[1] Organização Pan-Americana da Saúde (2018). Recomendações assistenciais para prevenção, diagnóstico e tratamento da hemorragia obstétrica.

[2] Alves AL, Francisco AA, Osanan GC, Vieira LB (2020). Postpartum hemorrhage: prevention, diagnosis and non-surgical management. Rev Bras Ginecol Obstet.

[3] Fujimori E, Sato AP, Szarfarc SC, Veiga GV, Oliveira VA, Colli C (2011). Anemia in Brazilian pregnant women before and after flour fortification with iron. Rev Saúde Pública.

[4] Shander A, Isbister J, Gombotz H (2016). Patient blood management: the global view. Transfusion.

[5] Shaylor R, Weiniger CF, Austin N, Tzabazis A, Shander A, Goodnough LT (2017). National and international guidelines for patient blood management in obstetrics: a qualitative review. Anesth Analg.

[6] Alves AL, Nagahama G, Nozaki AM (2020). Surgical management of postpartum hemorrhage. Rev Bras Ginecol Obstet.

[7] Muñoz M, Peña-Rosas JP, Robinson S, Milman N, Holzgreve W, Breymann C (2018). Patient blood management in obstetrics: management of anaemia and haematinic deficiencies in pregnancy and in the post-partum period: NATA consensus statement. Transfus Med.

[8] Ubom AE, Begum F, Ramasauskaite D, Nieto-Calvache AJ, Oguttu M, Nunes I (2025). FIGO good practice recommendations on anemia in pregnancy, to reduce the incidence and impact of postpartum hemorrhage (PPH). Int J Gynaecol Obstet.

[9] World Health Organization (2016). WHO recommendations on antenatal care for a positive pregnancy experience.

[10] World Health Organization (2024). Guideline on haemoglobin cutoffs to define anaemia in individuals and populations.

[11] Pavord S, Daru J, Prasannan N, Robinson S, Stanworth S, Girling J (2020). UK guidelines on the management of iron deficiency in pregnancy. Br J Haematol.

[12] Breymann C, Honegger C, Hosli I, Surbek D (2017). Diagnosis and treatment of iron-deficiency anaemia in pregnancy and postpartum. Arch Gynecol Obstet.

[13] Daru J, Colman K, Stanworth SJ, De La Salle B, Wood EM, Pasricha SR (2017). Serum ferritin as an indicator of iron status: what do we need to know?. Am J Clin Nutr.

[14] Georgieff MK (2020). Iron deficiency in pregnancy. Am J Obstet Gynecol.

[15] Rahmati S, Delpishe A, Azami M, Hafezi Ahmadi MR, Sayehmiri K (2017). Maternal anemia during pregnancy and infant low birth weight: a systematic review and meta-analysis. Int J Reprod Biomed.

[16] Levy A, Fraser D, Katz M, Mazor M, Sheiner E (2005). Maternal anemia during pregnancy is an independent risk factor for low birthweight and preterm delivery. Eur J Obstet Gynecol Reprod Biol.

[17] Lozoff B, Beard J, Connor J, Barbara F, Georgieff M, Schallert T (2006). Long-lasting neural and behavioral effects of iron deficiency in infancy. Nutr Rev.

[18] Surbek D, Vial Y, Girard T, Breymann C, Bencaiova GA, Baud D (2020). Patient blood management (PBM) in pregnancy and childbirth: literature review and expert opinion. Arch Gynecol Obstet.

[19] Finkelstein JL, Cuthbert A, Weeks J, Venkatramanan S, Larvie DY, De-Regil LM (2024). Daily oral iron supplementation during pregnancy. Cochrane Database Syst Rev.

[20] Tolkien Z, Stecher L, Mander AP, Pereira DI, Powell JJ (2015). Ferrous sulfate supplementation causes significant gastrointestinal side-effects in adults: a systematic review and meta-analysis. PLoS One.

[21] Stoffel NU, Cercamondi CI, Brittenham G, Zeder C, Geurts-Moespot AJ, Swinkels DW (2017). Iron absorption from oral iron supplements given on consecutive versus alternate days and as single morning doses versus twice-daily split dosing in iron-depleted women: two open-label, randomised controlled trials. Lancet Haematol.

[22] Stoffel NU, Zeder C, Brittenham GM, Moretti D, Zimmermann MB (2020). Iron absorption from supplements is greater with alternate day than with consecutive day dosing in iron-deficient anemic women. Haematologica.

[23] Kamath S, Parveen RS, Hegde S, Mathias EG, Nayak V, Boloor A (2024). Daily versus alternate day oral iron therapy in iron deficiency anemia: a systematic review. Naunyn Schmiedebergs Arch Pharmacol.

[24] Gomez-Ramirez S, Brilli E, Tarantino G, Munoz M (2018). Sucrosomial ® iron: a new generation iron for improving oral supplementation. Pharmaceuticals (Basel).

[25] Parisi F, Berti C, Mando C, Martinelli A, Mazzali C, Cetin I (2017). Effects of different regimens of iron prophylaxis on maternal iron status and pregnancy outcome: a randomized control trial. J Matern Fetal Neonatal Med.

[26] Antoine E, Mehedintu C, Mitran M, Diculescu D (2023). Sucrosomial® iron effectiveness in recovering from mild and moderate iron-deficiency anemia in the postpartum period. BMC Pregnancy Childbirth.

[27] (2021). Anemia in Pregnancy: ACOG Practice Bulletin, Number 233. Obstet Gynecol.

[28] Achebe MM, Gafter-Gvili A (2017). How I treat anemia in pregnancy: iron, cobalamin, and folate. Blood.

[29] Van Wyck DB, Martens MG, Seid MH, Baker JB, Mangione A (2007). Intravenous ferric carboxymaltose compared with oral iron in the treatment of postpartum anemia: a randomized controlled trial. Obstet Gynecol.

[30] Milman N (2012). Postpartum anemia II: prevention and treatment. Ann Hematol.

[31] Seid MH, Derman RJ, Baker JB, Banach W, Goldberg C, Rogers R (2008). Ferric carboxymaltose injection in the treatment of postpartum iron deficiency anemia: a randomized controlled clinical trial. Am J Obstet Gynecol.

[32] Auerbach M, Macdougall I (2017). The available intravenous iron formulations: history, efficacy, and toxicology. Hemodial Int.

[33] Christoph P, Schuller C, Studer H, Irion O, Tejada BM, Surbek D (2012). Intravenous iron treatment in pregnancy: comparison of high-dose ferric carboxymaltose vs. iron sucrose. J Perinat Med.

[34] Avni T, Bieber A, Grossman A, Green H, Leibovici L, Gafter-Gvili A (2015). The safety of intravenous iron preparations: systematic review and meta-analysis. Mayo Clin Proc.

[35] Wolf M, Rubin J, Achebe M, Econs MJ, Peacock M, Imel EA (2020). Effects of iron isomaltoside vs ferric carboxymaltose on hypophosphatemia in iron-deficiency anemia: two randomized clinical trials. JAMA.

[36] Govindappagari S, Burwick RM (2019). Treatment of iron deficiency anemia in pregnancy with intravenous versus oral iron: systematic review and meta-analysis. Am J Perinatol.

[37] Associação Brasileira de Hematologia, Hemoterapia e Terapia Celular (2023). Consenso da Associação Brasileira de Hematologia, Hemoterapia e Terapia Celular sobre Patient Blood Management.

[38] Main EK, Goffman D, Scavone BM, Low LK, Bingham D, Fontaine PL (2015). National partnership for maternal safety: consensus bundle on obstetric hemorrhage. Obstet Gynecol.

[39] Begley CM, Gyte GM, Devane D, McGuire W, Weeks A, Biesty LM (2019). Active versus expectant management for women in the third stage of labour. Cochrane Database Syst Rev.

[40] World Health Organization (2025). Consolidated guidelines for the prevention, diagnosis and treatment of postpartum haemorrhage.

[41] Widmer M, Piaggio G, Nguyen TM, Osoti A, Owa OO, Misra S (2018). Heat-stable carbetocin versus oxytocin to prevent hemorrhage after vaginal birth. N Engl J Med.

[42] WOMAN Trial Collaborators (2017). Effect of early tranexamic acid administration on mortality, hysterectomy, and other morbidities in women with post-partum haemorrhage (WOMAN): an international, randomised, double-blind, placebo-controlled trial. Lancet.

[43] Khan KS, Moore PA, Wilson MJ, Hooper R, Allard S, Wrench I (2017). Cell salvage and donor blood transfusion during cesarean section: a pragmatic, multicentre randomised controlled trial (SALVO). PLoS Med.

[44] Leffert L, Butwick A, Carvalho B, Arendt K, Bates SM, Friedman A (2018). The society for obstetric anesthesia and perinatology consensus statement on the anesthetic management of pregnant and postpartum women receiving thromboprophylaxis or higher dose anticoagulants. Anesth Analg.

[45] Kozek-Langenecker SA, Ahmed AB, Afshari A, Albaladejo P, Aldecoa C, Barauskas G (2017). Management of severe perioperative bleeding: guidelines from the European Society of Anaesthesiology: First update 2016. Eur J Anaesthesiol.

[46] Sarode R, Milling TJ, Refaai MA, Mangione A, Schneider A, Durn BL (2013). Efficacy and safety of a 4-factor prothrombin complex concentrate in patients on vitamin K antagonists presenting with major bleeding: a randomized, plasma-controlled, phase IIIb study. Circulation.

[47] Goldstein JN, Refaai MA, Milling TJ, Lewis B, Goldberg-Alberts R, Hug BA (2015). Four-factor prothrombin complex concentrate versus plasma for rapid vitamin K antagonist reversal in patients needing urgent surgical or invasive interventions: a phase 3b, open-label, non-inferiority, randomised trial. Lancet.

[48] Franchini M, Lippi G (2010). Prothrombin complex concentrates: an update. Blood Transfus.

[49] Collins PW, Cannings-John R, Bruynseels D, Mallaiah S, Dick J, Elton C (2017). Viscoelastometric-guided early fibrinogen concentrate replacement during postpartum haemorrhage: OBS2, a double-blind randomized controlled trial. Br J Anaesth.

[50] Escobar MF, Nassar AH, Theron G, Barnea ER, Nicholson W, Ramasauskaite D (2022). FIGO recommendations on the management of postpartum hemorrhage 2022. Int J Gynaecol Obstet.

[51] Green L, Knight M, Seeney FM, Hopkinson C, Collins PW, Collis RE (2016). The epidemiology and outcomes of women with postpartum haemorrhage requiring massive transfusion with eight or more units of red cells: a national cross-sectional study. BJOG.

[52] Levy JH, Goodnough LT (2015). How I use fibrinogen replacement therapy in acquired bleeding. Blood.

[53] Charbit B, Mandelbrot L, Samain E, Baron G, Haddaoui B, Keita H (2007). The decrease of fibrinogen is an early predictor of the severity of postpartum hemorrhage. J Thromb Haemost.

[54] Snegovskikh D, Souza D, Walton Z, Dai F, Rachler R, Garay A (2018). Point-of-care viscoelastic testing improves the outcome of pregnancies complicated by severe postpartum hemorrhage. J Clin Anesth.

[55] Zaidi A, Kohli R, Daru J, Estcourt L, Khan KS, Thangaratinam S (2020). Early use of fibrinogen replacement therapy in postpartum hemorrhage-a systematic review. Transfus Med Rev.

[56] Carson JL, Stanworth SJ, Dennis JA, Fergusson DA, Pagano MB, Roubinian NH (2025). Transfusion thresholds and other strategies for guiding red blood cell transfusion. Cochrane Database Syst Rev.

[57] Meybohm P, Froessler B, Goodnough LT, Klein AA, Muñoz M, Murphy MF (2017). "Simplified International Recommendations for the Implementation of Patient Blood Management" (SIR4PBM). Perioper Med (Lond).

[58] Shander A, Javidroozi M, Ozawa S, Hare GM (2011). What is really dangerous: anaemia or transfusion?. Br J Anaesth.

[59] Rajagopalan S, Mascha E, Na J, Sessler DI (2008). The effects of mild perioperative hypothermia on blood loss and transfusion requirement. Anesthesiology.

[60] Mueller MM, Van Remoortel H, Meybohm P, Aranko K, Aubron C, Burger R (2019). Patient blood management: recommendations from the 2018 Frankfurt Consensus Conference. JAMA.

[61] Le Bas A, Chandraharan E, Addei A, Arulkumaran S (2014). Use of the "obstetric shock index" as an adjunct in identifying significant blood loss in patients with massive postpartum hemorrhage. Int J Gynaecol Obstet.

[62] Rhodes A, Evans LE, Alhazzani W, Levy MM, Antonelli M, Ferrer R (2017). Surviving sepsis campaign: international guidelines for management of sepsis and septic shock: 2016. Intensive Care Med.

[63] Shields LE, Wiesner S, Fulton J, Pelletreau B (2015). Comprehensive maternal hemorrhage protocols reduce the use of blood products and improve patient safety. Am J Obstet Gynecol.

[64] Egenberg S, Oian P, Bru LE, Sautter M, Kristoffersen G, Eggebo TM (2015). Can inter-professional simulation training influence the frequency of blood transfusions after birth?. Acta Obstet Gynecol Scand.

[65] Leahy MF, Hofmann A, Towler S, Trentino KM, Burrows SA, Swain SG (2017). Improved outcomes and reduced costs associated with a health-system-wide patient blood management program: a retrospective observational study in four major adult tertiary-care hospitals. Transfusion.

[66] Meybohm P, Herrmann E, Steinbicker AU, Wittmann M, Gruenewald M, Fischer D (2016). Patient blood management is associated with a substantial reduction of red blood cell utilization and safe for patient's outcome: a prospective, multicenter cohort study with a noninferiority design. Ann Surg.

[67] Osanan GC, Padilla H, Reis MI, Tavares AB (2018). Strategy for zero maternal deaths by hemorrhage in Brazil: a multidisciplinary initiative to combat maternal morbimortality. Rev Bras Ginecol Obstet.

